# Ever at the ready for events that never happen

**DOI:** 10.1080/20008198.2017.1309934

**Published:** 2017-04-10

**Authors:** Jos F. Brosschot

**Affiliations:** ^a^ Institute of Psychology, Health, Medical and Neuropsychology Unit, Leiden University, RB Leiden, The Netherlands

**Keywords:** Perseverative cognition, unconscious stress, default stress response, generalized unsafety, perceived safety, loneliness

## Abstract

Stress, whether daily stress, work stress or traumatic stress, is unhealthy. This lecture covers three recent theoretical approaches in explaining the mechanisms underlying the influence of psychological stress on somatic health. It is argued that stress research should focus less on stressors themselves and put more emphasis on prolonged stress responses. Three mechanisms are identified that cause this unhealthy prolonged stress response: first, the partly-proven mechanism of perseverative cognition; second, the mechanism of unconscious stress, which is currently being explored; and third, the notion of the stress response being a default response that is inhibited only when safety is perceived. All three mechanisms are deeply rooted in millions of years of our evolution. Although the dangers of the past have virtually disappeared, many of us remain ever at the ready for events that never happen.

## 1. Introduction: long and short stress responses

### 1.1. Stress is a killer

Psychosocial stress, which I will hereafter call ‘stress’, is a killer. It has been classed as a worldwide epidemic by the World Health Organization and, according to various scientific sources, half of all sick days are taken due to stress and around two-thirds of visits to the doctor are stress related (Blaug, Kenyon, & Lekhi, 2007; Cartwright & Cooper, 2011; Cox, Griffiths, & Rial-Gonzalez, 2000; Pikhart & Pikhartova, 2015; Wilkinson & Marmot, 2003). If we look at cardiovascular disorders, 25% of the most stressed people are twice as likely to suffer a heart attack (Rosengren et al., 2004). These risks are comparable or even higher than those of the more traditional risk factors associated with heart disease like smoking and obesity (Blaug et al., 2007; Cartwright & Cooper, 2011; Cox et al., 2000). Traumatic stress is associated with increased morbidity and mortality rates (Boscarino, 2008; Flood et al., 2010; Gradus et al., 2015). High work stress increases your chances of developing cardiovascular disease four-fold, whereas caring for a partner with Alzheimer doubles that chance. Being in a difficult marriage can lead to a three times higher chance of developing heart problems. Long-term anxiety disorders are associated with a 2–7 times increased chance of cardiovascular disease (Bosma, Peter, Siegrist & Marmot, 1998; Chandola et al., 2008; Kiecolt-Glaser, McGuire, Robles, & Glaser, 2002; Kubzansky & Kawachi, 2000; Orth-Gomer et al., 2000; Pikhart & Pikhartova, 2015; Roest, Martens, de Jonge, & Denollet, 2010; Rosengren et al., 2004; Krantz & McCeney, 2002; Searle & Bennett, 2001; Vitaliano et al., 2002.

During my time working with the University of Amsterdam, and supported by the Royal Netherlands Academy of Arts and Sciences (KNAW), I angered test subjects not only to increase their blood pressure but also to study what determined how long this blood pressure stayed high (Brosschot, 1999; Brosschot & Thayer, 1998, 1999; Dorr, Brosschot, Sollers III & Thayer, 2007), because it is *prolonged* or *chronic* stress responses that ultimately damage our health (Brosschot, Pieper, & Thayer, 2005; Linden, Earle, Gerin, & Christenfeld, 1997; McEwen & Seeman, 1999; Pieper & Brosschot, 2005; Ursin, 1978; Ursin & Eriksen, 2004). Zebras do not develop gastric ulcers because they do not suffer from chronic stress, according to the stress scientist Robert Sapolsky (2004). Chronic stress seems to be a human invention.

True chronic stress responses are *daily* stress responses. They are continuous or, at the very least, last many hours at a time for many months or even years. Short-term stress responses are virtually harmless; being worried for a while, being scared, feeling disappointed or being on edge for a short amount of time cause a reaction in the body where blood pressure increases and adrenaline, ACTH and cortisol hormones are released into the blood. This is a completely natural and healthy bodily reaction. A short-term stress response *can* be fatal for people who are already physically fragile (e.g. through having a heart condition) if coupled with a very strong emotion. Short but extreme stress responses to traumatic events can also lead to long-term health problems (Wahlström, Michélsen, Schulman, Backheden, & Keskinen-Rosenqvist, 2013; Zaetta, Santonastaso, & Favaro, 2011; see also Olff, 2012) but, on the whole, short-term stress responses are not dangerous. On the contrary, they are completely natural responses to a threat. In ancient times, we would be dealing with bears, tigers or enemy tribes; these days we can be stressed from receiving an insult or starting an argument, misplacing a wallet or missing a train, having a flat tyre or reading a Facebook message (fear of missing out; FOMO).

Since Walter Cannon’s work around 100 years ago, this response in humans and animals has been called the fight-or-flight response, the biological part of which is evolutionary ancient (Cannon, 1915). The importance of our evolutionary background cannot be emphasized enough here. It probably comes as no surprise that we humans share 90% of our genes with chimpanzees. But did you know we share 85% of our genes with cows, and 73% with the zebra fish? Even 65% with chickens! (We share almost 40% of our genes with parasitic worms, and a quarter with grapes) (‘Introduction to bioinformatics’, 2016) These genes determine, partly, the same stress responses.

We thus share that fight-or-flight stress response with most animals. Chickens, apes and salmon all show stress responses similar to those of humans. Even oysters show an increase in the hormones ACTH and noradrenaline when they are stressed (exposed to prolonged shaking; Lacoste, Jalabert, Malham, Cueff, & Poulet, 2001). A short-term stress response is useful.

### 1.2. Human stress: long-term preparation with no action

A fight-or-flight response, with its increased heart rate and blood pressure, results in what seems similar to physical effort responses (Lovallo, 2005): what you get when lift weights or go running for example. Your heart reacts to an average stressor, e.g. an argument, the same way as it would if you ran up several flights of stairs. There is one important difference: during a stressor, like an argument, we humans only *prepare* ourselves for fighting or fleeing rather than raising a fist or running away. Our heart rate increases, just like an athlete’s heart rate does before a 100 m sprint (which incidentally almost doubles).

A brief *preparation* for fighting or fleeing is not harmful. A little stress can be a good thing for all kinds of performances, like having an interview or giving a formal speech. This is often called ‘eustress’, or good stress. It is *prolonged* stress responses that are potentially unhealthy.

Most stressful events, i.e. stressors, do not last long. An unpleasant comment at work is over in a second, and a few minutes bickering with a partner ends quickly. An important job interview rarely lasts more than an hour or so. The daily encounter with an intimidating boss or receiving a dreaded tax assessment: these are all extremely brief stressors. *Too* brief in fact to explain the prolonged unhealthy stress response. How can we explain the prolonged stress response? My colleagues and I have spent the last 15 years creating three hypothetical explanations for this, all of which I will be laying out for you here.

In short, the first hypothesis concerns the endless worrying and brooding that takes place between stressful events: negative rumination. This is something that has been studied extensively by us and by others. The second hypothesis concerns unconscious stress which forms part of our current ongoing research. And the third hypothesis I will reveal to you at the end of my lecture, as this one still finds itself in the rather more speculative corner. First, let us return briefly to that strong stress response called anger.

### 1.3. It’s all in the mind

It is not such an easy thing to make people angry, while keeping within the ethical norms of scientific research. One thing we would do was get the test subject to attempt a difficult puzzle while constantly pestering him or her with snide remarks such as ‘oh, just give up, this isn’t working’. Or my favourite, ‘hold your arm still, we’re trying to measure your blood pressure’. This did not work at all, especially as the students conducting the experiment were far too friendly. Could we have expected anything less from psychology students! They much prefer helping people and making them happy than trying to anger them. When it finally worked with one test subject they got so angry they complained at the faculty and not long after this an ethical committee was established in the psychology department in Amsterdam.

In the anger research done by us and by others (Brosschot, 1999; Brosschot & Thayer, 1998, 1999; Dorr et al., 2007; Glynn, Christenfeld, & Gerin, 2002; Schwartz, Gerin, Davidson, & Christenfeld, 2000; Schwartz et al., 2003), it was noted time and again that test subjects’ blood pressure would return to normal quicker if the person being tested could do something nasty back to the one who had angered them. This included giving small electric shocks or criticizing that person on a performance evaluation form. Venting frustrations on someone or something *else* does not work: throwing bottles against a wall or slamming a fist into a pillow is fruitless. Frustrations, it seems, need to be dealt with at their core: revenge must be taken on the one causing the frustration. Interestingly, it is not even necessary to perform the revenge or retaliation acts as it were: as long as the *option is there*, the blood pressure will quickly return to normal. It is *all in the mind*.

We were however interested in the issue of what kept blood pressure so high without the opportunity for retaliation, an opportunity which of course we do not normally have. Was it normal for it to stay so high? We compared the effects of angering subjects with physical exercise (cycling on a stationary bicycle). We did this for the same duration it took to get angry and we made sure the blood pressure increased to the same level. Blood pressure returned to normal much quicker from the cycling than it did after the test subject was angered. In fact, it was often still high even when the test subject admitted to no longer being angry. Why? What was going on?

The issue was clarified by research done by a colleague of ours in New York, Bill Gerin. Gerin also angered his test subjects, but he then did something else: he distracted half of them with busy colourful posters and other eye-catching objects. This group’s blood pressure lowered significantly quicker than that of the not-distracted group: distraction worked. The not-distracted group’s blood pressure stayed high for a longer time not because the test subjects were still angry, but because they continued to *ruminate* angrily (Glynn et al., 2002; Schwartz et al., 2000, 2003). While Gerin limited this idea to *anger* and blood pressure *recovery*, Julian Thayer and I quickly saw the potential in this idea. We convinced Gerin that this angry rumination not only delays the recovery from a stress response but also can cause stress responses *before* the stressor even takes place due to our *anticipation* of this stressor: we are ruminating about it before it happens. We also believed that stress responses could arise unexpectedly, without the presence of stressors, when we thought about a stressor in our life. So not only could *recovery* and *anticipatory* reactions be explained by this rumination, but also all stress reactions lacking an immediate stressor. Voila, we had the explanation for prolonged stress responses. We coined this negative rumination and worrying as *perseverative cognition*, from which the *perseverative cognition hypothesis* was born (Brosschot, Gerin, & Thayer, 2006; Brosschot & Thayer, 2004). This brings me to the first hypothesis.

## 2. Perseverative cognition

Perseverative cognition is the ‘constant (*perseverative*) thinking (cognition) about negative events in the past or in the future’. Officially, the definition is ‘the cognitive activation of representations of stressors’. The wonderful thing about this is that it is a purely human stress theory. Animals do not spend time worrying about things, or so we presume: it is only humans that possess the parts of the brain which have the capacity to create representations of events in the past, i.e. memories, and representations of events that might happen in the future. This has brought with it an enormous evolutionary advantage for humans: we were better able to learn from the past and make plans for the future. But the drawback of all that capacity to think and ponder about the past and the future is that it can often give rise to anxious worrying. And, usually, this worry concerns things that do not even happen. Our bodies thus react to stressful events that do not take place.

Meanwhile we found it strange that, in over 60 years of stress research, so little had been done in regard to the causes of prolonged stress responses. This is most probably because a vast deal of research is based on experiments concerning animals and, as we know, animals do not ruminate. This is the reason why chronic stress is a human invention.

Since the publication of this perseverative cognition hypothesis with Gerin and Thayer, there has been a lot of international research in the field. My research group showed, in the lab as well as in daily life, that negative rumination increases cardiac activity. In one of those studies, student Eduard van Dijk and I gave 80 volunteers a small box to carry with them for a day which measured their heart activity. We asked them to keep a diary noting their ruminations and worries throughout the day: the stressors they experienced. Heart activity indeed increased thanks to worry. It was not possible to link this increased heart activity to negative emotions nor to smoking, drinking coffee or physical exercise; it linked overwhelmingly to rumination. Doctoral candidate Suzanne Pieper, financed by The Netherlands Organisation for Scientific Research (NWO), later reproduced these results (Brosschot, van Dijk, & Thayer, 2007; Pieper, Brosschot, van der Leeden, & Thayer, 2007).

Other research groups have come to similar conclusions, for example the research done by Cristina Ottaviani from Rome. Together with us she has shown in a recent meta-analysis that all the research collectively points to the conclusion that perseverative cognition leads to increased physiological activity, not only of the cardiovascular system but also of our hormone system (Ottaviani et al., 2016b). The journal that published this meta-analysis, *Psychological Bulletin*, had previously rejected the original article on the perseverative cognition hypothesis exactly 10 years previously. It was considered then to be ‘too speculative’. We find the recent publication a satisfying reparation. Many studies have shown that excessive worrying can increase the risk of illness in the long term (Broadbent, Petrie, Alley, & Booth, 2003; Brosschot & van der Doef, 2006; Fortune et al., 2003; Gerin et al., 2012; Holman et al., 2008; Jellesma, Verkuil, & Brosschot, 2009; Radstaak, Geurts, Beckers, Brosschot, & Kompier, 2014; Thayer. & Brosschot, 2010; Tully, Cosh, & Baune, 2013; Van Laethem et al., 2015; Verhoeven et al., 2009; Verkuil et al., 2011; Verkuil & Brosschot, 2016; Verkuil, Brosschot, Gebhardt, & Korrelboom, 2015; Verkuil, Brosschot, Gebhardt, & Thayer, 2010; Verkuil, Brosschot, Meerman, & Thayer, 2012).

### 2.1. Worry interventions

It is not easy to stop worrying. Current ‘anti-worrying’ methods are not in optimal working order so we are currently trying two new methods. Doctoral candidate Anke Versluis is attempting to lessen the worry and lower the heart activity of people who suffer from high work stress unconsciously. She does this by sending mindfulness-type tasks to their smartphones multiple times a day. Andreas Burger, financed by means of a Netherlands Foundation for Scientific Research (NWO) VENI grant to Bart Verkuil, is attempting to break the vicious circle of worry (cognitive perseveration) through subtly influencing the brain by stimulating a nerve end in the ear (Burger et al., 2016). The results of these studies are yet to be revealed.

### 2.2. Why perseverative cognition?

Perseverative cognition: why choose such a difficult phrase? Why choose a new phrase altogether? Why not just ‘worrying’ or ‘rumination’? At first, I could not even pronounce it in English, which incidentally is somewhat ironic for something you have thought of yourself.

The reason for choosing such an umbrella term is that there are many more stress related thought activities than just worrying. Pondering, brooding, agonizing over something or ruminating (‘peinzen’ and ‘piekeren’ in Dutch), all fall under the ‘perseverative cognition’ category. People think long and hard about various problems without necessarily ‘worrying’ about them but it can still have physiological effects, as was discovered by Verkuil (Verkuil, Brosschot, Borkovec, & Thayer, 2009).

But there is more. Our mind likes to wander and we spend large parts of the day daydreaming. The psychologists Killingsworth and Gilbert discovered (Killingsworth & Gilbert, 2010) that we spend half our waking hours daydreaming, of which around 40% is about positive things and the rest about neutral or negative things. Surprisingly enough, daydreaming about positive things did *not* make their test subjects any happier, while daydreaming about negative *as well as* neutral things made people *less* happy. Thus, daydreaming makes you unhappy. They did not call their *Science* article ‘A wandering mind is an unhappy mind’ for no reason. Over the years, Ottaviani has convincingly shown that part of daydreaming is formed of perseverative cognition, and that this part goes hand-in-hand with a stronger and unhealthier bodily activity (Ottaviani et al., 2016a; Ottaviani & Couyoumdjian, 2013; Ottaviani, Medea, Lonigro, Tarvainen, & Couyoumdjian, 2015; Ottaviani et al., 2015; Ottaviani, Shapiro, & Couyoumdjian, 2013).

### 2.3. Not all prolonged stress responses are explained

Can perseverative cognition explain all prolonged stress responses? We started to realize in the last seven years that there is more to it. We discovered that, after a day of worrying, heart activity remained high during sleep (Brosschot et al., 2007) and not only during REM sleep in the latter part of the night. This was also discussed by researchers in the US and Japan (Sakakibara, Kanematsu, Yasuma, & Hayano, 2008; Weise, Ong, Tesler, Kim, & Roth, 2013; Yoshino & Matsuoka, 2009). Tica Hall found a strong indication of this in her sleep laboratory in Pittsburgh (Hall et al., 2004). She had a group of students sleep in her laboratory, of which half were told before they went to sleep they had to get up at 8 am to deliver a speech to an audience. The other half were told nothing. In the latter group, heart activity decreased as per normal during the night. But in the first group, heart activity remained high and did so for the whole duration of the night: not because they slept any worse, which of course was to be expected, but because apparently their brains and bodies were constantly engaged with stressing about the looming speech. Traumatic events – experienced a long time ago – may suddenly show up during sleep, in the form of a nightmare, in PTSD patients, known to be accompanied by EEG sleep abnormalities terrors (de Boer, Nijdam, Hofman, Olff, & Talamini, 2013).

Why is this so important? It is important because we spend around a third of our lives asleep and if the prolonged stress response stays ‘on’ even during the night when we are sleeping it means a serious prolonged stress response: we are not only alert throughout the day, but throughout the night too.

Another reason why this is so important is that a sleeping human cannot consciously be worrying. The obvious question here is how is it possible to have increased heart activity from the events of the day before, when you are asleep and thus cannot consciously be worrying? Do stressful feelings and thoughts continue into the night at an unconscious level? There is indeed some proof for this in neurobiological sleep research (Walker & Stickgold, 2004; Walker & van der Helm, 2009).

It also raised another question. If unconscious stress-related cognition (unconscious stress) is active at night and can lead to physiological effects, could this possibly happen during the day too? This brings me to the second hypothesis: the unconscious stress hypothesis (Brosschot, 2010; Brosschot, Verkuil, & Thayer, 2010).

## 3. Unconscious stress

Pieper measured the heart activity of over 100 teachers in their daily life and asked them, via palmtop computers, if they had been worrying/ruminating. As expected, rumination caused elevated heart activity, but heart activity remained high for an average of two hours after the test subjects had stopped worrying. This continuation could not be explained by stressors, emotions, physical movement, smoking or anything else. It seemed that the test subjects continued to worry without even knowing it themselves. We consulted available literature to see if this could be possible and if we could find any further leads into the physiological effects of unconscious stress. We had positive results in both cases (Pieper, Brosschot, van der Leeden, & Thayer, 2010).

One person who tested the physiological effects of unconscious stress was Rebecca Levy from Toronto. She wanted to see if it was possible to influence the stress response of older people by subliminally (and thus unconsciously) presenting them with negative words related to growing old i.e. stressful stimuli for older people. Negative words were confused, sick, demented and frail. She compared this with positive words such as wise, mature and experienced. Levy discovered that blood pressure indeed increased when test subjects were exposed to the negative words, and that it stayed lower if they had been exposed to the positive words (Levy, Hausdorff, Hencke & Wiel, 2000).

One of the things we have realized about the brain during the last 20 years is that we are not conscious of most of its activity (Bargh & Morsella, 2008; Dijksterhuis & Nordgren, 2006; Kihlstrom, 1987). It therefore seemed highly plausible that neither are we conscious of a large part of stress-related information in the brain, while it can still have visible physiological effects. This meant we had to expand the perseverative cognition hypothesis. Our bodies not only react to stressful events that do not take place, but we are not even conscious of our bodies doing so.

Verkuil, Thayer and I published the new theory about unconscious stress (Brosschot, 2010; Brosschot et al., 2010), and we have also more recently published some evidence in support. We have found further evidence of the physiological effects of subliminally presented threatening stimuli in other studies, among which is doctoral candidate Melanie van der Ploeg’s work (Garfinkel et al., 2016; van der Ploeg, Brosschot, Thayer, & Verkuil, 2016).

How do you *measure* unconscious stress in day to day life? How do you measure something in humans that they are not aware of? We are currently researching this with the financial support of a TOP grant from the Netherlands Organisation for Health Research and Development (ZonMW). I cannot do justice in my lecture today to the theoretical and methodical complexity of our studies, but I can report an example from one of our findings. We were able to measure unconscious negative emotion using an ingenious method by Marcus Quirin from Osnabrück University (Quirin et al., 2016) both with van der Ploeg in the Leiden laboratory and doctoral candidate Mirjam Radstaak in Nijmegen. This unconscious negative emotion was associated with slower blood pressure recovery after a stressor (Brosschot et al., 2014).

Research on unconscious stress continues at a slow but steady pace. Dare we say that we have solved the puzzle: that all prolonged stress responses are due to conscious and unconscious stress related cognition but that the latter is not fully measurable? No. I found this explanation unsatisfying and a bit too easy.

Furthermore, chronic stress responses often seem to appear when they are clearly not caused by *stress*, nor by *unconscious stress*. Take for example loneliness. Loneliness often goes hand-in-hand with a chronic physiological response that looks very much like a stress response. It also carries increased chance of illness or early death (Cacioppo, Cacioppo, Capitanio, & Cole, 2015; Coan, Shaver, & Mikulincer, 2010; Hawkley, Burleson, Berntson, & Cacioppo, 2003; Porges, 2004, 2007; Steptoe, Shankar, Demakakos, & Wardle, 2013; Yang, Boena, Gerkena, Schorpp & Mullan Harris, 2016), and it is something that is seen more frequently in our individualistic and greying society. But what causes the stress response in loneliness? In itself it is not a threat, it is not a stressor. It does seem however that those who suffer from loneliness are lacking in something: a warm, supportive, safe social network – something which is so important for social animals such as humans; call it love for a better word. But why would one show signs of a stress response, even a chronic stress response, in the absence of love if there is no sign whatsoever of a stressor? This brings me to my third and final hypothetical explanation: the role of safely and the default stress response when safety is not present (Brosschot, Verkuil, & Thayer, 2016a, 2016b).

## 4. Not stress but safety? The default stress response

When a starling does not have access to water to clean its wings, it becomes more vigilant and more aware of danger. The starling who cannot wash its wings, even when they are not necessarily dirty, seems therefore to show a stronger stress response while there is no sign of an actual stressor (Brilot & Bateson, 2012). A similar thing happens with zebra finches (Krause & Ruploh, 2016) who show a higher level of the stress hormone corticosterone if they have not been able to bathe for a while. This should come as no surprise for, without water to wash one’s wings, fleeing when danger approaches is a far less effective operation: one cannot take flight as quickly with dirty wings. Being more alert when there are no washing opportunities is therefore highly beneficial for the survival of these birds; the more vigilant creatures were the ones who could pass on their genes.

In another example, a squid who had lost part of one of its eight legs would regard a small neutral object (e.g. a wire) as threatening. When presented with such an object it retreated quicker, hid, changed position or squirted a cloud of ink quicker than before disfigurement (Crook, Dickson, Hanlon, & Walters, 2014). Thus, a mildly handicapped squid is much more alert even when there is no immediate threat.

How can the lack of washing facilities or missing part of one of your eight legs lead to seeing threats when there are none? And these are most certainly not isolated cases. Farmers could tell you that dirty and badly cared for animals are more stressed, and it has emerged in many studies that animals do not need a direct threat (a stressor) to become stressed. All that is needed is the presence of another (strange) animal or human, an owner’s unusual behaviour, a freshly cleaned litter box or nowhere to hide yourself, for stress levels to increase (Arrant, Schramm-Sapyta, & Kuhn, 2013; Ellis, 2009; Fischer, Franco, & Romero, 2016; Miura, Qiao, & Ohta, 2002; Petty, Kramer, & Larrison, 1996; van den Buuse, van Acker, Fluttert, & de Kloet, 2001; e.g. ‘Understanding animals’, 2016). Typically, patients with posttraumatic stress disorder reply to all kinds of internal or external cues as if they encounter trauma. They show a response of acute threat while the current threat level is practically zero.

Stress responses when there are no direct threats nor specific obvious dangers is something very common. Why is this so? The answer is simple but surprising. A stress response does not need a stressor at all, it is simply always ‘on’, and it stays on, as long as there is no obvious safety. It turns ‘off’ if the situation or surroundings are perceived as safe and turns on again if this perceived safety disappears. The stress response is therefore a ‘default’ response. Default means a preselected condition where there is no other input i.e. no relevant information. The relevant information in regard to a stress response is safety. With no safety signal the stress response stays on: everything falls back to default and is seen as unsafe. The default here is a state of generalized unsafety.

We humans are naturally, by default, afraid of the unknown. As we grow up we learn to recognize the safety signals but, from the very beginning we fear without a sign of threat. This is wonderfully portrayed in a Sigmund strip in the Volkskrant newspaper where a patient complains about being ‘scared of the unknown’ to the doctor. ‘I don’t know’, answers the man when doctor Sigmund asks him what exactly he is scared of, ‘I don’t know anything about it’.

Even so, this idea of a default stress response does sound strange, and what is worse is that this goes in stark contrast to current stress theories. Stress theories speak of a stress response as a *response* to a *stressor* i.e. as a direct response to a threat.

The idea of a default stress response does correspond to modern evolutionary theoretical insights about stress, anxiety, PTSD and, more importantly, to neurobiological knowledge: the knowledge of how the brain and the nervous system works. The parts of the brain responsible for the stress response, for example the amygdala, are chronically suppressed by the prefrontal cortex (Ahern et al., 2001; Amat et al., 2005; Fragkaki, Thomaes, & Sijbrandij, 2016; Grupe & Nitschke, 2013; Kubala, Christianson, Kaufman, Watkins, & Maier, 2012; Lanius, Frewen, Tursich, Jetly, & McKinnon, 2015; Maier, 2015; Motzkin, Philippi, Wolf, Baskaya, & Koenigs, 2015). As Steve Maier and colleagues have demonstrated (Amat et al., 2005; Kubala et al., 2012; Maier, 2015), this prefrontal suppression only takes place when the brain has perceived safety. If safety is no longer perceived, the pressure is immediately eased, the brake is immediately lifted, the amygdala resumes its activity and the body is instantly ready to flee or stand and fight. Heart activity increases and blood pressure goes up.

The stress response is therefore always set to high alertness but kept suppressed as long as safety can be perceived and let go whenever it is no longer safe. This neurobiological principle is very common and is known as the *Hughlings Jackson principle*. In 1884, Hughlings Jackson wrote that evolutionary old responses like the stress response are not actually turned on or pushed into action but rather let go or released (Hughlings Jackson, 1884). That release happens much quicker than turning something on, which is extremely important with such a vital response mechanism.

High alertness and an ultrafast stress response when in unsafe surroundings has enormous survival power. In the wild it is better to play it safe and to sprint away from something 10 times too often than once too few. A principle known as the *better safe than sorry principle* in evolutionary theory. Creatures who waited to see what exact dangers were approaching did not survive and thus did not reproduce. Those who sprinted, flew or swam away at the first sign of danger continued to thrive (Nesse, 2005; Trimmer, Paul, Mendl, McNamara, & Houston, 2013).

## 5. GUTS

We have developed the idea that a stress response is a default response that is chronically suppressed if there is no speak of safety, into a new theory. We have called this theory the Generalized Unsafety Theory of Stress, otherwise known as GUTS. Verkuil, Thayer and I have extensively argued this theory in a number of articles (Brosschot et al., 2016a, 2016b), the latest of which has just been published in *Neurobiology and Biobehavioral Reviews*. GUTS takes us to entirely new hypotheses.

### 5.1. The heavier you are the more dangerous your world

There are many bodily conditions in which we see increased physiological activity such as increased heart activity, high blood pressure and an increase of the so-called stress hormones, which look very similar to prolonged stress responses: for example obesity, low aerobic fitness and old age (Hansen, Johnsen, Sollers, Stenvik, & Thayer, 2004; Julius, 1995; Thayer, Yamamoto, & Brosschot, 2010; Zulfiqar, Jurivich, Gao & Singer, 2010). These conditions also carry significant health risks but they are not often considered to be direct causes of stress responses. We think that people in these conditions are stressed. We think that, irrespective of other biological mechanisms that cause the increase in their physiological activity, the default stress response in the above physical conditions is not being fully suppressed because, through millions of years of evolution, they carry with them a less adequate fight or flight response. They are ‘not optimally resilient’ bodily conditions. We call them *compromised*. An older or less fit body reacts slower to its potentially dangerous surroundings, like the starling or the squid, which means their world is less safe. The heavier or less fit you are, the more dangerous your world is. High alertness was therefore key for survival. For millions of years, our world was a predatory and dangerous place to live in, each day could be your last. Therefore, in summary, our bodies do not necessarily react to stressful events, but rather cease reacting when safety is perceived, or when safety would have been perceived by our ancestors.

I am approaching the end of my lecture. We have come a long way in stress research in the last century, from when George M. Beard (1881) claimed the following about the rise of *Nervousness* (an old term for stress) 135 years ago:

The chief and primary cause of … [the] very rapid increase of nervousness in modern civilization, [is due to] these five characteristics: steam power, the periodical press, the telegraph, the sciences, and the mental activity of women.

That was 1881. Nowadays we have stressors from wholly new sources: work, relationships, social media, FOMO. I hope that I have made clear my belief that there should be less focus on stressors themselves and more emphasis on prolonged stress responses. I have spoken about three mechanisms that cause this unhealthy prolonged stress response in which I plan to further my research: first, the partly-proven perseverative cognition; second, the unconscious stress, which we are currently working on; and third, the default stress response, for which we are currently developing research ideas. All three are deeply rooted in millions of years of evolution. Although those ancient dangers have essentially disappeared, many of us remain ever at the ready for events that never happen.
